# Correlation between chest CT scores and clinical impact in patients re-infected with COVID-19 during the two attacks: an observational study

**DOI:** 10.1186/s43055-022-00739-7

**Published:** 2022-03-07

**Authors:** Mona Mohammed Fatouh, Nour Mohamed Kandil, Nermeen Mahmoud EL Garhy

**Affiliations:** Radiology Department, Faculty of Medicine, Cairo University, Giza, Egypt

**Keywords:** COVID-19, CT chest, Re-infection, CT severity score

## Abstract

**Background:**

Worldwide, millions of people got COVID-19 infection since the start of the pandemic with a large number of deaths. Re-infection with SARS-CoV-2 is possible, because it can mutate into new strains as it is an RNA virus. The main objective of our study is to correlate between CT severity score of the patients re-infected with COVID-19 during the first and second attack and its clinical impact.

**Results:**

We performed a retrospective cohort study. It was carried out on fifty symptomatic patients (11 females and 39 males). Their ages ranged from 38 to 71 years. We included only patients who were re-infected after more than 6 months of the first infection and showed clinical symptoms with SARS-CoV-2 PCR-positive test. We found that CT severity score was decreased in the second infection in 47 (94%) of our patients associated with decreased respiratory distress as well as oxygen requirements, while the CT severity score was increased in two patients and only one patient showed no change in CT score severity between two infections.

**Conclusion:**

The reduction in CT severity score in the majority of re-infected patients suggested the role of the immunity developed from first infection in protection against severe lung affection in case of repeated infection even after 6 months despite poor immunity against re-infection.

## Background

Since the origin of COVID-19 in Wuhan, China, at the end of 2019, it was reported that it had infected over 38 million person and caused at least one million deaths worldwide [1]. It is an RNA virus, so it has the ability to mutate. SARS-CoV-2 showed variable genetic composition in different geographical areas [2]. Many cases re-infected with COVID-19 have been documented since August 2020.

A robust immune response could be developed in the persons recovered from COVID-19 to clear the virus. In spite of this, it is not clearly known if the initial infection gives a protective immunity against recurrent infections. Although it was reported in some recent studies that presence of COVID-19 antibodies from previous infection could give protection against re-infection in most of individuals, re-infection could occur in certain cases [3]. In spite of the presence of antibodies, it is possible that re-infection occurs with different strains of human corona viruses [4]. 

Till now, re-infection with COVID-19 and reactivation were documented only as case reports [5, 6] and based upon genotyping of the viral strains in both attacks of infection. Depending on genotyping in the confirmation of re-infection is not a practical approach as it is not suitable for clinical application during the care of infected patients and it could not be applied on a large scale [7].

We assessed patients in an integrated health care system that had a recurrent positive SARS-CoV-2 test 6 months after the first positive one to avoid this gap.

Nowadays, COVID-19 diagnosis is based on reverse transcriptase polymerase chain reaction (RT-PCR) testing. Samples collected commonly through oropharyngeal or nasopharyngeal swabs. The RT-PCR testing has variable sensitivity depending on the site from which samples are collected [8]. Because of this, its results could be falsely negative in a significant number of cases, raising the need for another diagnostic tool to accurately detect patients with COVID-19 infection [9]. 

Chest computed tomography (CT) suggested from researches from areas with high numbers of COVID-19 infected cases is to be used as an additional diagnostic tool of COVID-19 infection beside the PCR test. Guidelines in the UK do not support routine use of CT in assessment of COVID-19 patients [10]. But, The Royal College of Radiologists demonstrated situations in which early CT imaging in patients with suspected COVID-19 will be suitable clinically [10], especially among hospitalized patients highly suspected to have COVID-19 infection but their RT-PCR test is negative and showed inconclusive other diagnostic modalities, such as chest X-ray [11]. 

Bilateral and multifocal ground glass opacities are the most common and typical CT features of COVID-19 pneumonia. The most typical location is peripheral, posterior and basal parts of the lungs [12]. Fine reticulations, peri-broncho-vascular thickening, vascular dilatations within pneumonia areas, or architectural distortion are other findings that have been reported [13]. Other findings as micronodules, excavations, septal lines, mediastinal lymph node enlargement, or pleural effusions usually are not detected [14]. 

COVID-19 pneumonia has similar appearance in CT chest as that of other viral pneumonitis. The differentiating points are: In COVID-19 pneumonitis the lesions show mainly peripheral location, they can affect the five lobes, and that peri-broncho-vascular thickening or fine atelectasis could be seen more frequently [15]. In contrary, influenza pneumonia showed increased prevalence of nodular or micronodular tree-in-bud pattern and may be associated with pleural effusions [16]. 

We aimed at studying the differences in clinical symptoms and CT findings in patients re-infected with COVID-19 after 6 months of the initial infection, as well as comparing the CT severity score of each patient between the two episodes of infection.

## Methods

A retrospective cohort study was carried out on fifty symptomatic patients (11 females and 39 males). Their ages ranged from 38 to 71 years. All patients were referred to the diagnostic radiology department from chest clinic from April 2020 to August 2021. In this study, COVID-19 confirmed cases were those who presented by clinical symptoms of COVID-19 and their RT-PCR test was positive based on their nasopharyngeal or oropharyngeal specimens. We included only patients who were re-infected more than 6 months after the first infection and showed clinical symptoms, SARS-CoV-2 PCR-positive test and underwent CT examination of chest in the two infections. The clinical and demographic information of patients is recorded and followed in a comprehensive electronic system in our institution that record the clinical and laboratory data of the patients from April 2020 to August 2021. The protocol related to this study has been proposed and approved in the ethics committee of our University.

Patients without positive PCR test for the two episodes of infection were excluded from our study.

### CT Chest parameters

Multislice CT chest was done to all patients using 16 channels MSCT (Siemens). Reconstructed axial, coronal and sagittal images were done to all patients; also complementary mediastinal images were taken.

The CT Scanning parameters were as follows: slice thickness = 1–2 mm, FOV = 350 mm × 350 mm, tube rotation = 0.6–0.9 s and detector collimation = 1 mm. The irradiation dose parameters were as follows: 120–130 kvp and 100–200 mA (according to the machine type as well as patient age and weight). Intravenous contrast administration was not utilized.

### CT chest image analysis

We reviewed and compared the two CT scans of each patient independently by two expert radiologists, who were blinded to the clinical severity information and the final decision was taken with consensus. The following CT features were analyzed in each patient:

- *Ground glass opacity (GGO)* is the non-specific hazy opacification of the lung in the X-ray or computed tomography with no obliteration of bronchial or vascular markings [17].
- *Consolidation* defined as an area of increased attenuation
  o which obscures the bronchial and vascular markings[17].
- *Atoll sign* represents an area of GGO surrounded by near complete ring of consolidation [17].
- *“Bull’s-eye sign” or “Target sign” or “Double-halo”* Central nodule or consolidation and peripheral organization rim with ground glass in between [18].
- *“Crazy-paving pattern”* represent thickened interlobular septa superimposed on GGO [17].
- *Nodule with ground glass halo* A nodule is an opacity less than 3 cm in diameter with regular or irregular outline. In general, viral pneumonitis is characterized by the presence of nodules. The reported incidence of pulmonary nodules in patients with COVID-19 pneumonia is 3–13% and may be associated with surrounding halo [17].
- *Peri-lobular fibrosis*.
- *Laterality of the lung opacities* (a) unilateral, (b) bilateral parenchymal lung affection with opacities
- *Distribution of the lung opacities* (a) peripheral (involving most the outer 1/3 of the lungs), (b)
- central (involving most the inner 2/3 of the lungs), or (c)
- both central and peripheral (no definite clear predominance).
- *Affected lobes* upper or lower
- *Other findings* were also reported such as (a) pleural effusion (b) mediastinal lymphadenopathy.

### CT severity score system

Affected lung in percentage per lobe (max. 25 points): (0% = 0 points), (< 5 = 1 point), (5–25% = 2 points), (25–50% = 3 points), (50–75% = 4 points), (75–100% = 5 points) [19]. 

### Statistical analysis

Data was coded and entered using the statistical package for the Social Sciences (SPSS) version 26 (IBM Corp., Armonk, NY, USA). Data was summarized using mean, standard deviation, median, minimum and maximum in quantitative data and using frequency (count) and relative frequency (percentage) for categorical data. Comparisons between quantitative variables were done using the nonparametric Mann–Whitney test. For comparison of serial measurements within each patient, the nonparametric Wilcoxon signed rank test was used [20]. For comparing serial categorical data, McNemar Test was used [21]. *P* values less than 0.05 were considered as statistically significant.

## Results

This study included 50 patients (11 females 22%, 39 males 78%) with age ranged from 38 to 71 years (mean age 55.26 years), suffering from different symptoms of COVID-19. Thirty-eight patients were suffering from chronic illness. The count and percentage of the associated chronic diseases of the examined patients are mentioned in Table 1.

**Table 1:** The count and percentage of the associated chronic diseases of the examined patients

	Count	%
*Associated findings and chronic diseases*		
SLE	1	2.0
Renal	2	4.0
RA	1	2.0
IHD	2	4.0
Hypertensive	5	10.0
Hepatic	4	8.0
Diabetic	10	20.0
Cardiac	7	14.0
Cancer	4	8.0
Autoimmune	2	4.0
No	12	24.0

Quantitative variables of the age, CT severity score and O_2_ saturation of initial infection and re-infection were presented in Table 2 as mean with standard deviation. At the initial infection, CT score was 14.72 and O_2_ saturation was 86.8 while in the re-infection CT score was 8.36 and O_2_ saturation was 90.8.

**Table 2:** The maximum, minimum, mean, median and standard deviation (SD) of the age, initial CT score and re-infection CT score

	Mean	Standard deviation	Median	Minimum	Maximum
Age	55.26	8.63	55.00	38.00	71.00
CT score	14.72	4.60	16.00	4.00	20.00
CT score (re-infect)	8.36	4.02	8.00	2.00	18.00

### Clinical evaluation of the patients in the initial and repeated attacks of infection

We assessed the patients as regard respiratory distress and we found that, in the initial infection 80% of patients presented by respiratory distress and required O_2_, while in the second episode of infection the percent of patients suffering from distress decreased to 74%.

Other frequently detected symptoms were fever and myalgia. They presented by 100% of patients cough during the first infection. In the repeated attack, fever and myalgia remain the most prevalent symptoms but their frequency decreased to 96% (Table 3).

**Table 3:** Comparison between clinical symptoms presented during initial infection and re-infection

	First infection		Re-infection		*P* value
	Count	%	Count	%	
*Anosmia*					
Yes	36	72.0	33	66.0	0.690
No	14	28.0	17	34.0	
*Taste*					
Yes	23	46.0	16	32.0	0.230
No	27	54.0	34	68.0	
*Fever*					
Yes	50	100.0	48	96.0	0.500
No	0	0.0	2	4.0	
*Cough*					
Yes	44	88.0	30	60.0	0.003
No	6	12.0	20	40.0	
*Respiratory distress*					
Yes	40	80.0	37	74.0	0.453
No	10	20.0	13	26.0	
*GIT (Diarrhea)*					
Yes	23	46.0	16	32.0	0.210
No	27	54.0	34	68.0	
*Myalgia*					
Yes	50	100.0	48	96.0	0.500
No	0	0.0	2	4.0	
*PCR*					
Yes	50	100.0	50	100.0	–

### Radiological evaluation

We found that ground glass opacification was the most common pattern of lung affection in both episodes of infection which was detected in 100% of the examined patients. Consolidation was seen in 60% of patients during the first episode and its incidence decreased 28% in the second attack (Table 4).

**Table 4:** Showed the radiological CT chest finding during initial infection and re-infection

	First infection		Re-infection		*P* value
	Count	%	Count	%	
*Unilateral/Bilateral*					
Unilateral	0	0.0	3	6.0	0.250
Bilateral	50	100.0	47	94.0	
*Upper/Lower lobes*					
Lower	49	98.0	50	100.0	1
Both	1	2.0	0	0.0	
*Ground glass opacities*					
Yes	50	100.0	50	100.0	–
*Consolidations*					
Yes	30	60.0	14	28.0	< 0.001
No	20	40.0	36	72.0	
*Mediastinal lymph nodes*					
Yes	22	44.0	25	50.0	0.678
No	28	56.0	25	50.0	
*Pleural effusions*					
Yes	0	0.0	3	6.0	0.250
No	50	100.0	47	94.0	

As regard the other important radiological signs, we reported the following:

*In the initial infection*

**Fig. 1.**
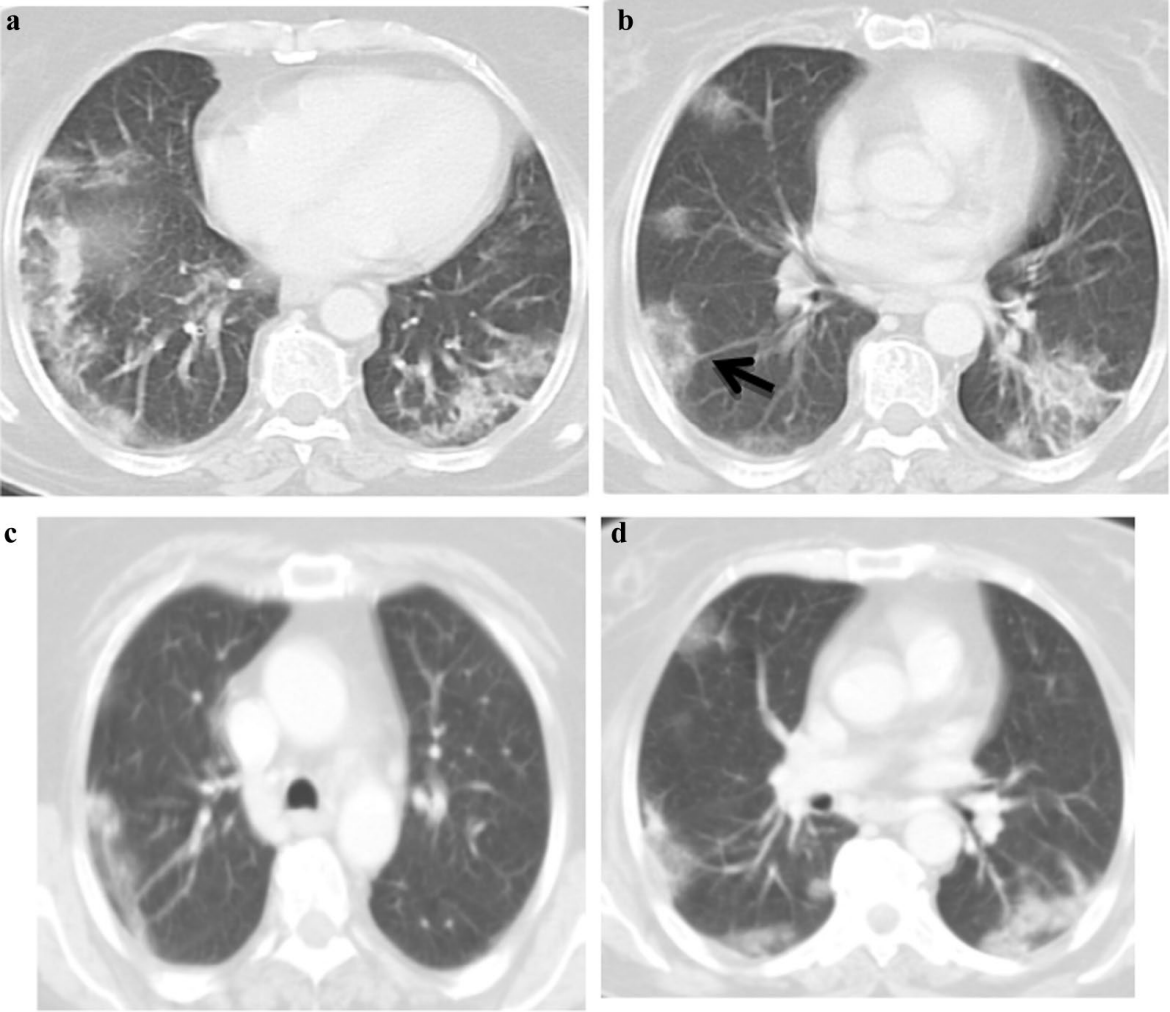
A 52-year-old female known history of cancer colon presented with anosmia, cough, fever, diarrhea and myalgia at September 2020 and positive PCR for COVID 19 infection, **a** and **b** post-contrast CT axial cuts shows bilateral patchy subpleural ground glass opacities with right lower lobe Atoll sign (arrowed). The patient diagnosed as COVID 19 infection with CT score severity: 10. Second infection dated March 2021 the patient presented with fever, cough, respiratory distress and myalgia with positive PCR test for COVID 19 infection. **c** and **d** postcontrast CT chest images show bilateral patchy subpleural ground glass opacities. The patient diagnosed as COVID 19 re-infection with CT severity score: 4

**Fig. 2.**
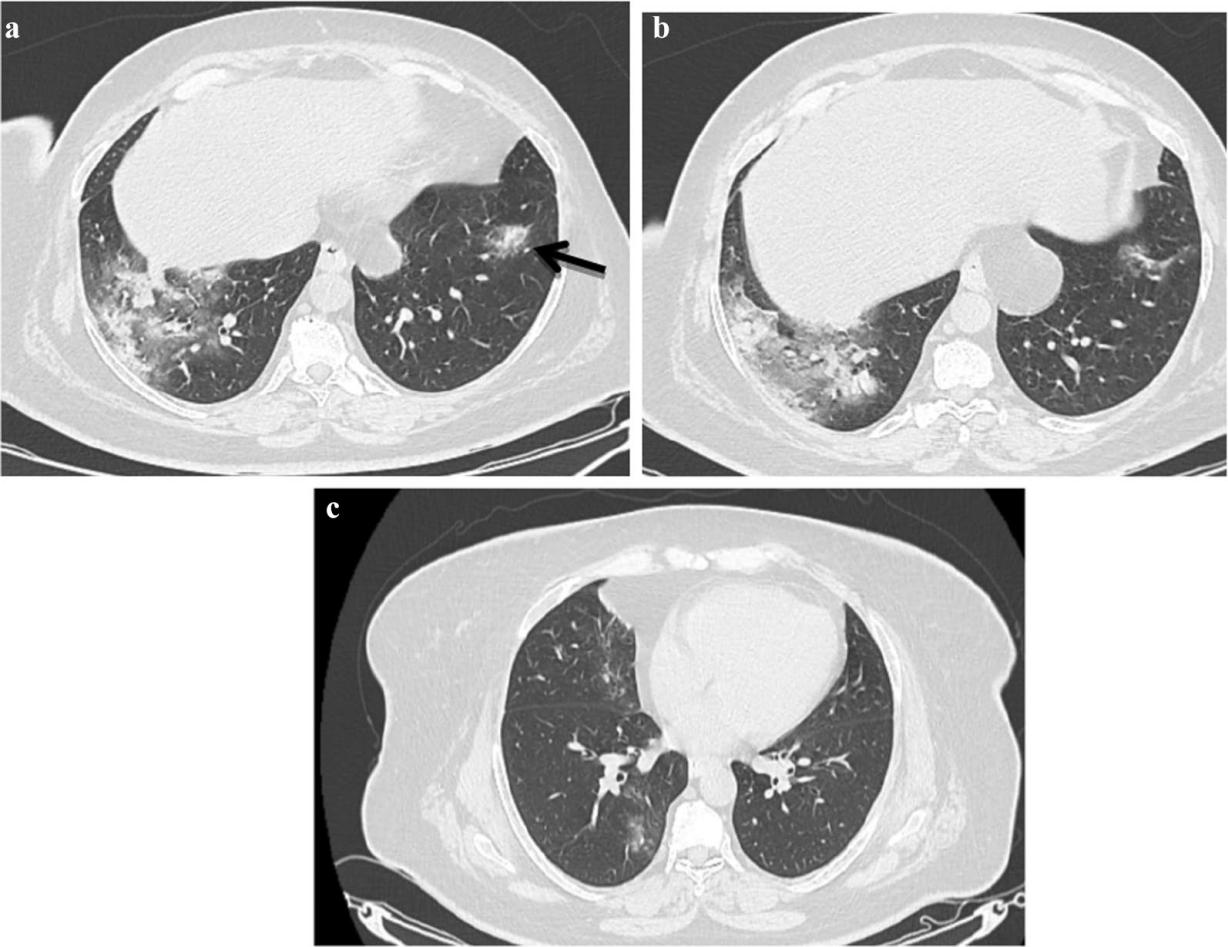
A 47-year-old female known history of rheumatoid arthritis presented with anosmia, loss of taste, fever, diarrhea and myalgia at May 2020 and positive PCR for COVID 19 infection, **a** and **b** noncontrast CT axial cuts shows bilateral patchy subpleural ground glass opacities and consolidations with left lower lobe nodule with ground glass halo sign noted. The patient diagnosed as COVID 19 infection with CT score severity: 16. Second infection dated June 2021 the patient presented with anosmia, loss of taste, fever, diarrhea and myalgia with positive PCR test for COVID 19 infection. **c** noncontrast CT chest images show bilateral patchy subpleural ground glass opacities. The patient diagnosed as COVID 19 re-infection with CT severity score: 5

- *Atoll sign* seen in 64% of patients (Fig. 1).
- *Crazy paving* seen in 70% of examined patients.
- *Nodule with ground glass halo* presented in 42% of examined patients (Fig. 2).
- *Bull’s eye sign, architectural distortion and peri-lobular fibrosis* not detected in any of our patients initially (Table 5).

**Table 5:** Radiological comparison between initial infection and re-infection as regard the presence of other important radiological signs

	First infection		Second infection		*P* value
	Count	%	Count	%	
*Atoll*					
Yes	32	64.0	18	36.0	0.011
No	18	36.0	32	64.0	
*Crazy paving*					
Yes	35	70.0	19	38.0	0.001
No	15	30.0	31	62.0	
*Bull's eye sign*					
Yes	0	0	7	14.0	0.016
No	50	100.0	43	86.0	
*Pei lobular fibrosis*					
Yes	0	0	9	18.0	0.004
No	50	100.0	41	82.0	
*Architectural distortion*					
Yes	0	0	6	12.0	0.031
No	50	100.0	44	88.0	
*Nodule with halo*					
Yes	21	42.0	8	16.0	0.004
No	29	58.0	42	84.0	

*In the re-infection*

**Fig. 3.**
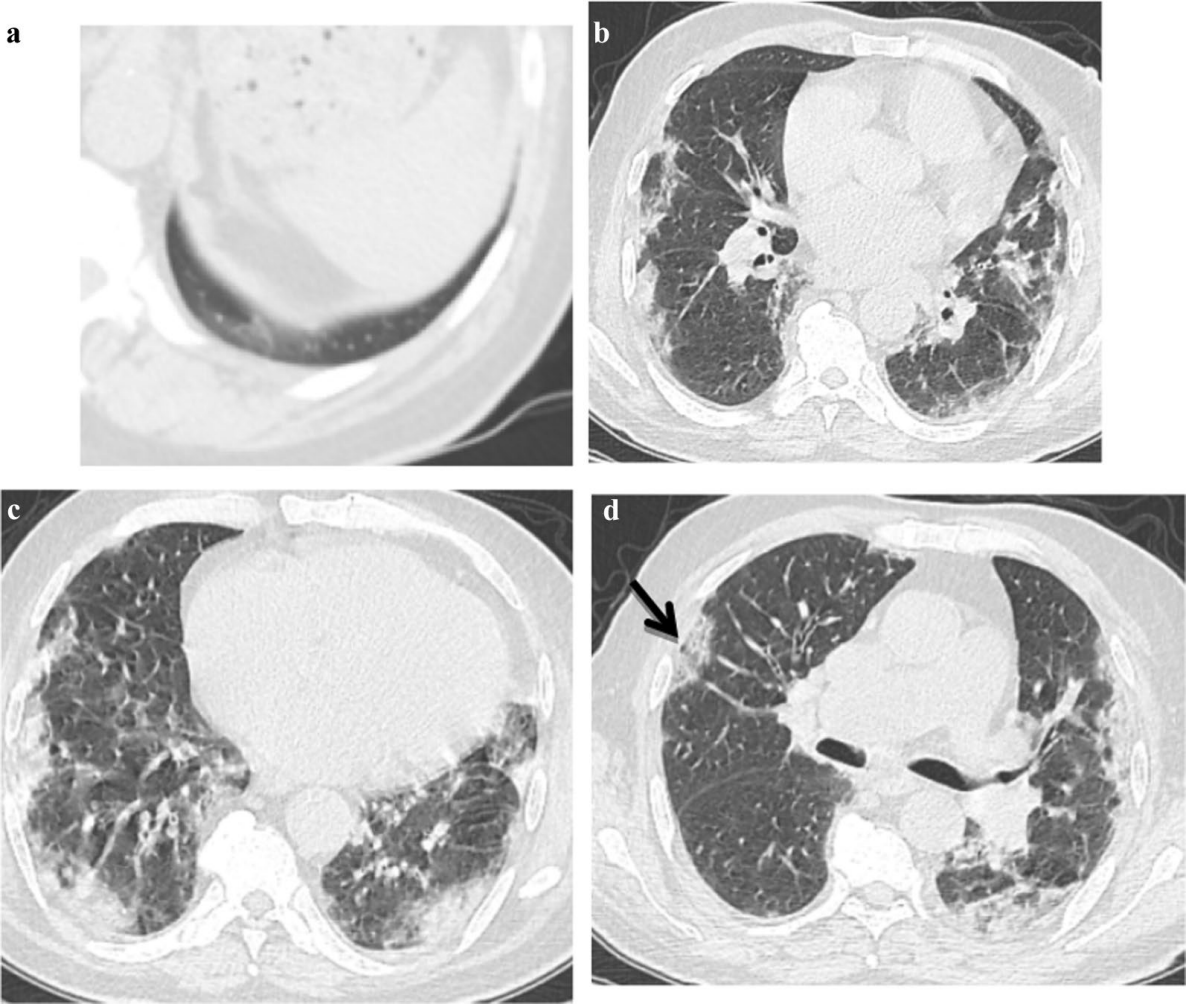
A 71-year-old male known cardiac presented with anosmia, fever and myalgia at March 2020 and positive PCR for COVID 19 infection, **a** noncontrast CT axial cuts shows bilateral few small subpleural faint ground glass opacities. The patient diagnosed as COVID 19 infection with CT score severity: 4. Second infection dated January 2021 the patient presented with fever, respiratory distress, cough and myalgia with positive PCR test for COVID 19 infection. **b**–**d** noncontrast CT chest images show bilateral patchy ground glass opacities with crazy paving sign (Arrowed) and consolidations. The patient diagnosed as COVID 19 re-infection with CT severity score: 18

- *Atoll sign* seen in 36% of patients.
- *Crazy paving* 38% of examined patients (Fig. 3).
- *Bull’s eye sign* 14% of patients.
- *Nodule with ground glass halo* 16% of patients
- *Architectural distortion* 12% of examined patients
- *Peri-lobular fibrosis* 18% of patients (Table 5).

Bilateral parenchymal lung affection with opacities in 100% of the examined patients in the first episode and in 94% of the second one (Table 4).

**Fig. 4.**
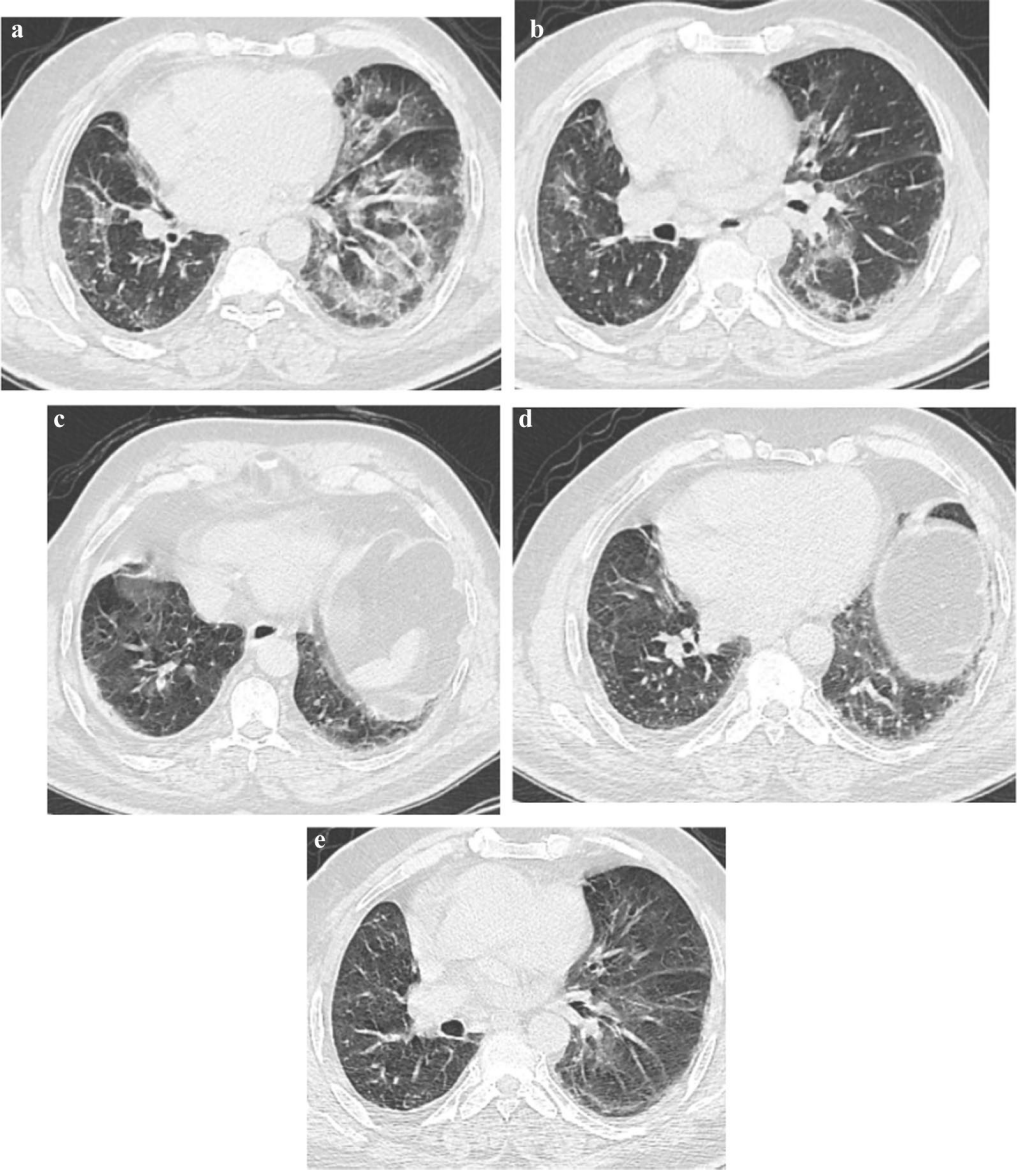
A 65-year-old female known IHD presented with anosmia, loss of taste, cough, fever, respiratory distress, diarrhea and myalgia at May 2020 and positive PCR for COVID 19 infection, **a** and **b** noncontrast CT axial cuts show bilateral faint ground glass opacities with crazy paving sign. The patient diagnosed as COVID 19 infection with CT score severity: 20. Second infection dated May 2021 the patient presented with fever, diarrhea and myalgia with positive PCR test for COVID 19 infection. **c**–**e** noncontrast CT chest images show bilateral patchy subpleural faint ground glass opacities and reticulations. The patient diagnosed as COVID 19 re-infection with CT severity score: 5

**Fig. 5.**
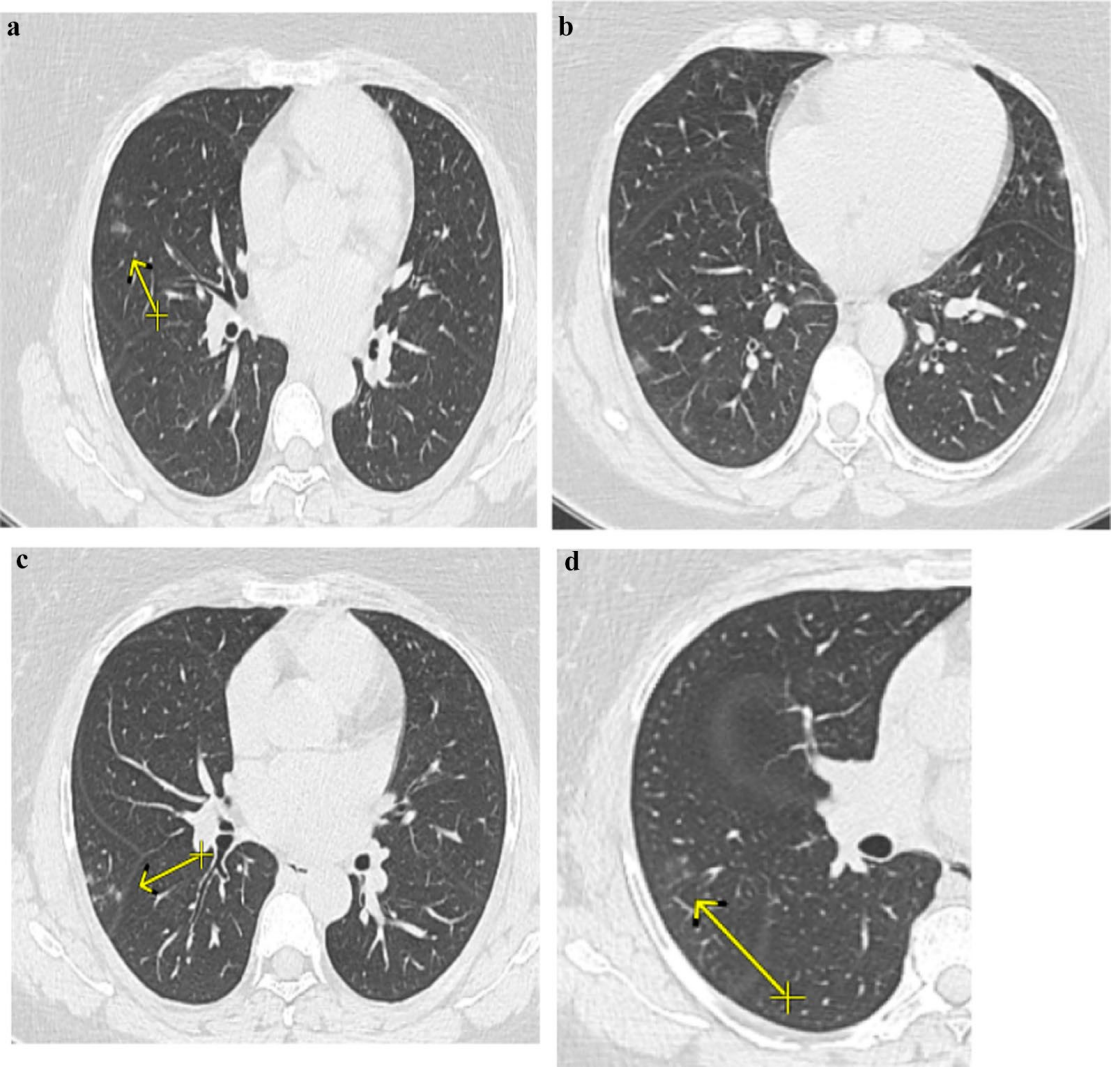
A 33-year-old male presented with anosmia, loss of taste, fever, diarrhea and myalgia at July 2020 and positive PCR for COVID 19 infection, **a** and **b** noncontrast CT axial cuts shows bilateral faint ground glass opacities. The patient diagnosed as COVID 19 infection with CT score severity: 6. Second infection dated June 2021 the patient presented with anosmia with positive PCR test for COVID 19 infection. **c** and **d** noncontrast CT chest images show right lower lobar patchy subpleural faint ground glass opacities. The patient diagnosed as COVID 19 re-infection with CT severity score: 2

Lower lobes were the most affected lobes in the initial examination of the examined patients. They were presented by 98%. In the re-infection, they were the only affected lobes in the re-infected examined and presented by 100% of patients (Table 4, Figs. 4 and 5).

*Other findings* mediastinal lymphadenopathy was seen in 44% of the cases in the initial infection and increased to 50% in the re-infection. Pleural effusion was not detected in the initial CT study but seen in 6% of patients in the second infection (Table 4).

### CT severity score

Forty-seven patients (94%) showed decreased CT score severity in the second episode, two patients (4%) showed increased CT score severity and only one patient (2%), Fig. 3 with no change in the CT score severity between the two infections. There was a statistical significant relation between the mean value of CT severity score of the re-infected COVID-19 patients (mean = 8.36) versus the initial COVID-19 infection (mean = 14.72), (*P* < 0.001) (Table 6).

**Table 6:** Showed a significant decrease in the CT severity score of the re-infected COVID-19 patients (mean = 8.36) versus the initial COVID-19 infection (mean = 14.72), (*P* < 0.001)

	Mean	Standard deviation	Median	Minimum	Maximum	*P* value
CT score	14.72	4.60	16.00	4.00	20.00	< 0.001
CT score (re-infect)	8.36	4.02	8.00	2.00	18.00	

**Fig. 6:**
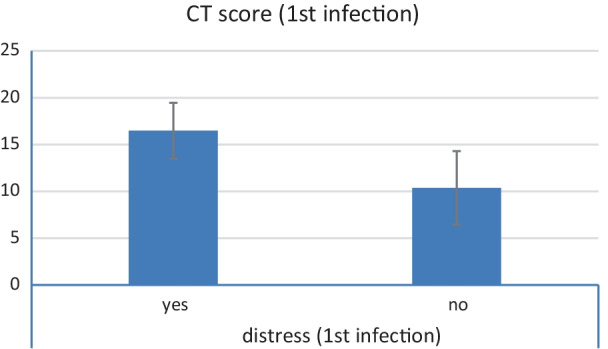
Relation between CT severity score and respiratory distress during initial infection

**Fig. 7:**
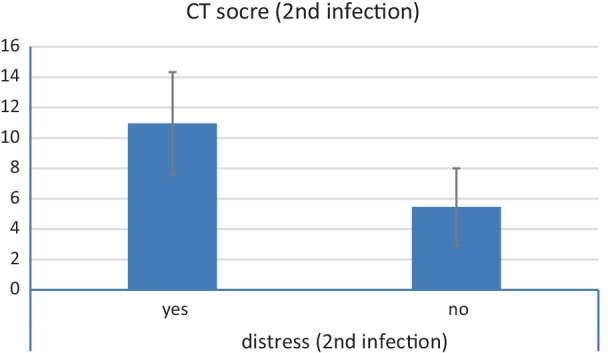
Relation between CT severity score and respiratory distress during second infection

We assessed the relation between CT severity score (expressing the radiological severity of COVID-19 infection) and the presence of respiratory distress on the one hand and the relation between CT severity score and reduction of O_2_ saturation (reflecting the clinical severity of COVID-19 infection) on the other hand, in both episodes of infection and we found that:

There was a statistically significant relation between CT severity score and respiratory distress in the initial infection as well as in the re-infection with *P* value less than 0.001 in both episodes (Tables 7 and 8 and Figs. 6 and 7).

**Table 7:** Relation between distress and CT severity score (during first infection)

	Distress (first infection)										
	Yes					No					*P* value
	Mean	SD	Median	Minimum	Maximum	Mean	SD	Median	Minimum	Maximum	
CT score (first infection)	16.48	2.99	16.50	10.00	20.00	10.38	3.93	8.00	6.00	16.00	< 0.001

**Table 8:** Relation between distress and CT severity score (during second infection)

	Distress (second infection)										
	Yes					No					*P* value
	Mean	SD	Median	Minimum	Maximum	Mean	SD	Median	Minimum	Maximum	
CT score (second infection)	10.97	3.37	10.00	5.00	18.00	5.46	2.54	5.00	2.00	10.00	< 0.001

**Fig. 8:**
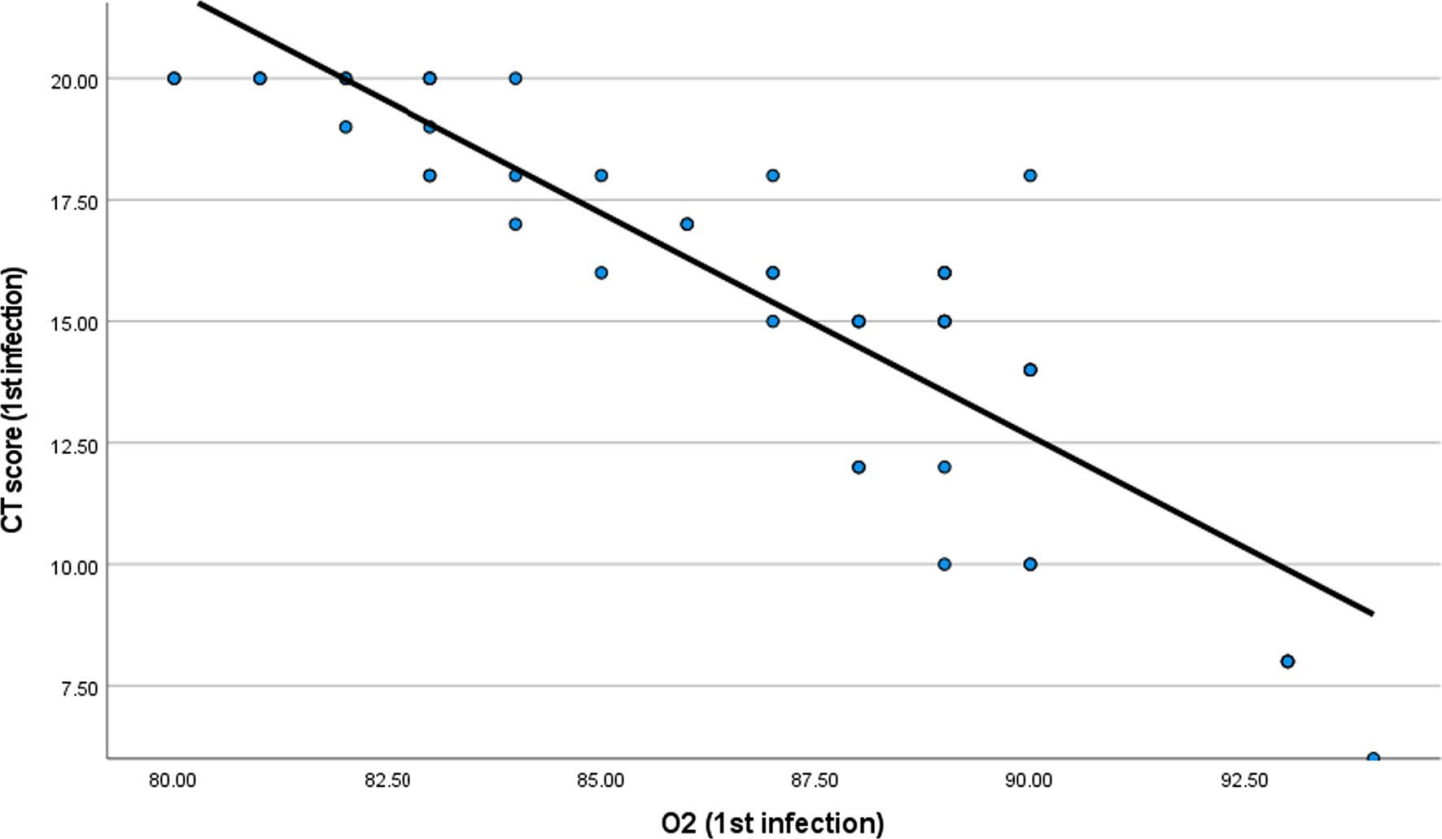
Relation between CT severity score and O_2_ saturation during first infection

**Fig. 9:**
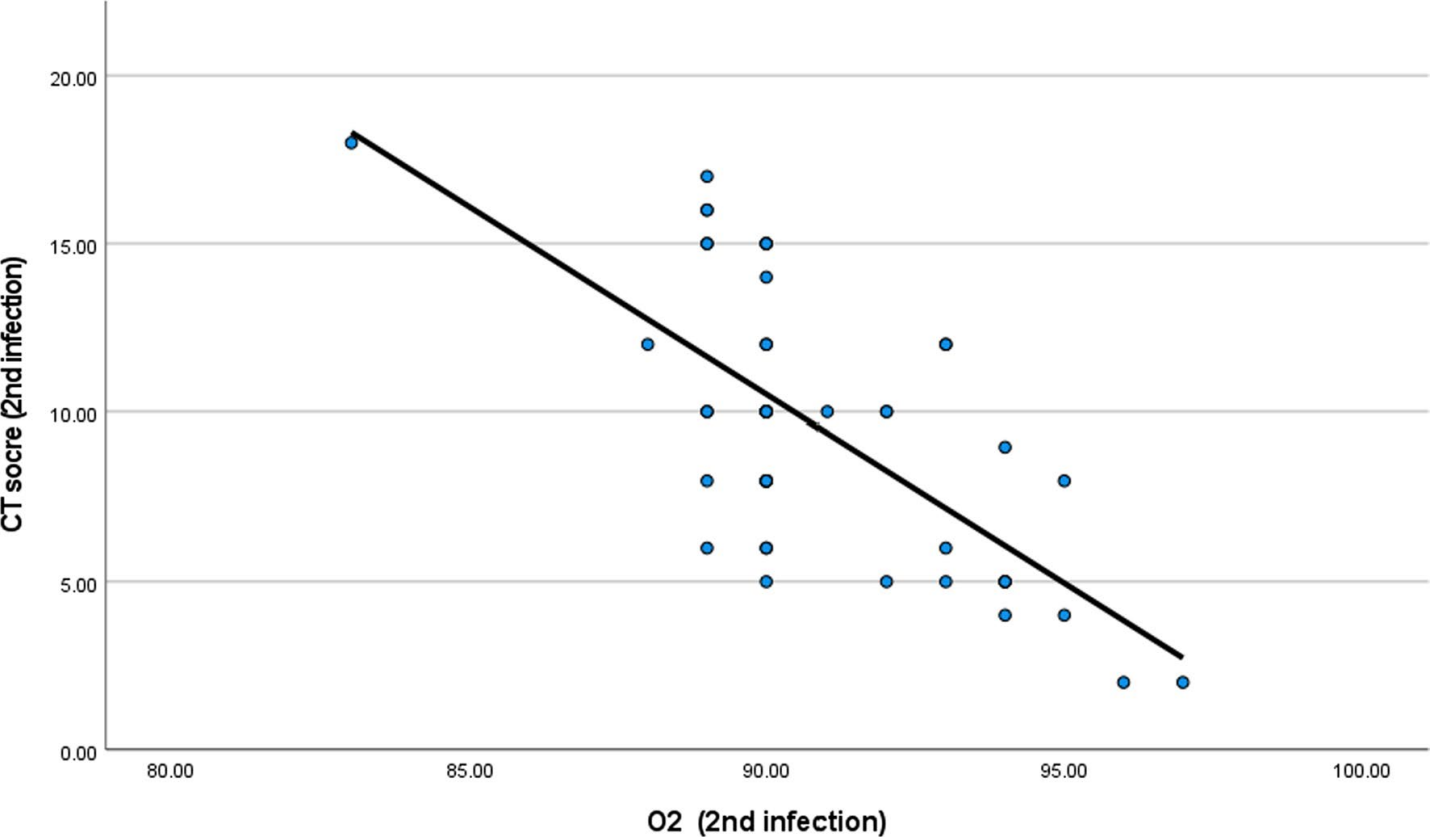
Relation between CT severity score and O_2_ saturation during first infection

There was a statistical significant relation between reduction of O_2_ and CT severity score in the first and second infections with *P* value less than 0.001 (Tables 9 and 10 and Figs. 8 and 9).

**Table 9:** Relation between CT severity score and O_2_ saturation (during first infection)

	O_2_ (first infection)
*CT score (first infection)*	
Correlation coefficient	− 0.887-
*P* value	< 0.001
*N*	50

**Table 10:** Relation between CT severity score and O_2_ saturation (during second infection)

	O_2_ (second infection)
*CT score (second infection)*	
Correlation coefficient	− 0.627-
*P* value	< 0.001
*N*	50

## Discussion

Immunological memory is formed for a pathogen when a person infected with it which leads to its imprint in the immune system. This immunological memory can protect that person from recurrent infection for decades. This is due to the formation of B and T lymphocytes with antigen-specific memory in addition to antibodies that protect against subsequent infections [22]. 

Viruses that cause systemic infections, such as rubella, mumps, measles, in addition to hepatitis A virus, is rare to cause re-infection. In contrast to this, viruses that cause mucosal infection without viremia, such as influenza, respiratory syncytial virus, and seasonal coronavirus, is common to recur [23]. This is explained by the much longer antibody response in systemic viral infections [24]. Recurrence of infection in respiratory viruses is caused either by weakened primary immune response such as in infection with respiratory syncytial virus, or exposure to genotype of another species (e.g., nasal viruses) or high diversity of viruses (e.g., flu viruses) [25]. 

Re-infection with COVID-19 has been reported worldwide [26]. Although zero conversion occurs in most of patients with SARS-COV-2 infection, the titer of binding and neutralizing antibodies varies between different patients and reduces with time [22]. Previous studies found that the more severe the disease the higher levels of neutralizing antibody titers formed and that these antibodies could be detected till 2–3 months after infection, in contrast to asymptomatic patients or patients with mild symptoms who had lower antibody titers that start to decline in less than 2 months from the beginning of the primary symptoms [27]. 

The antibodies titer required to neutralize the virus and to protect against recurrent infections should be known [28]. Researches done on SARS-CoV-2 and other coronaviruses reported that infection with coronaviruses can initiate long-term T-cell immunity which plays an important role in maintaining long-term immunity against viruses [21]. SARS-CoV-2-specific T, CD4 and CD8 cells reported in some studies to last more than 6 months after initial infection [24]. 

Susceptibility to re-infection depends on several factors that vary in different individuals including: the rate of antibody response and its durability as well as the duration of cellular immunity [25]. 

In our study we aimed at assessment of CT findings in patients with two episodes of infection with COVID-19 with more than 6 months gap between the two attacks to be sure that the level of neutralizing antibodies from the first episode has been decreased and the shedding of virus has stopped.

As regard the clinical assessment of the patients we reported that dyspnea was found in 80% of patients in the initial phase and decreased to 74% in the second phase. Other common symptoms were: fever and myalgia which presented by 100% of patients during the first infection but their frequency decreased to 96% in the re-infection.

Our reported data differed from what documented in another previous study in which the most common symptoms were headache (70.3%), loss of smell (70.2%), nasal obstruction (67.8%) and cough (63.2%) [29]. 

In another study that compared the symptoms experienced by patients re-infected with COVID between both infections, their results were similar to ours in the first attack of infection as they found that the most common symptoms were fever (63%) cough and dyspnea each was detected in (54%); moreover, they reported decrease in frequency of fever to 27% in the second episode, but they differed from us as they reported increased frequency of dyspnea to 72% in second episode [30]. 

As regard the radiological findings, no similar researches recorded and compared CT features in patients re-infected with COVID-19 since the beginning of world pandemic at December 2019 throughout first, second and third waves, most probably due to the previous thought that the immunity acquired through infection with COVID protects against re-infection. But with the progression of the pandemic and discovery of new strains of corona virus, as well as loss of the immunity acquired after first infection, the occurrence of re-infection become more common and reported.

We found two studies that compared CT findings of different patients between the first and second pandemic waves, one done in Ghana [31] and the other in Egypt [18]. In our study, we compared CT findings in the same patients that infected twice. The Egyptian study done by Samir et al. reported one case of COVID19 re-infection but they did not describe any details about his or her radiological findings in both attacks of infection.

In our research, we documented that the most common pattern of lung affection was ground glass opacification which was presented in (100%) of patients in both episodes. Bilateral lung affection was seen in 100% of patients in initial infection and decreased to 94% in re-infection. The most affected lobe is the lower lobe which detected in 98% in initial infection and in 100% of patients in re-infection.

This matched with what had been reported in the literature that the most typical pattern of COVID-19 pneumonia is ground glass opacification that was classically bilateral predominated in the peripheral and basal part of the lungs [12]. The frequent affection of lower lung zones is most probably due to the anatomy of the lower lung bronchus, which is short in length and thick, making the lower bronchus easy to be cached by the virus.

As regard the other less common radiological signs we detected that: Atoll sign seen in 64% of patients, Crazy paving seen in 70% of examined patients and nodule with ground glass halo presented in 42% of examined patients during the first attack, while in the re-infection the frequency of these signs was decreased.

Bull’s eye sign, architectural distortion and peri-lobular fibrosis are not detected in any of our patients initially. Peri-lobular fibrosis and architectural distortion develop late in the course of infection process and we assessed the patients in the acute phase. In the second phase, we detected bull's eye sign, architectural distortion and peri-lobular fibrosis in small percent of patients, the two later signs seen in the second phase may be due to sequel of the initial infection.

In the study done in Egypt [18], they reported some atypical CT findings which were only encountered during the second pandemic wave. They included the bronchiectatic changes, the “head-cheese” pattern, the “bulls-eye” sign and the cavitation.

As regard the severity of infection we used CT severity score, and we detected significant decrease in the CT severity score of the re-infected COVID-19 patients versus the initial COVID-19 infection. The decrease in CT severity score in most of patients in the recurrent infection is mostly due to the residual effect of the immune response developed after the initial infection which was not strong enough to prevent re-infection but it showed a role in decreasing disease severity.

In the Egyptian study [18], they reported 10% rise in the clinical and CT severity-score as well as the hospitalization rates in the second pandemic wave reported through the fourth and fifth decades of life.

We checked the relation between the radiological severity (assessed with CT severity score) and clinical severity expressed by suffering from respiratory distress and reduction of O_2_ saturation, and we reported a statistically significant relation between CT severity score and the presence of respiratory distress as well as reduction in O_2_ saturation in both episodes of infection.

This matched with the result of study done by Saeed et al. [32], who found that oxygen requirements increase with the increasing CT severity.

The main limitations to our study were small sample size, lack of availability of the genetic study which allows the comparison between the virus strains between the two episodes, as well as lack of other similar researches to be compared with our study.

## Conclusion

COVID-19 re-infection is possible. The severity of re-infection is variable and may cause death. This necessitates the constant use of masks, social distancing and other preventive measures in recovered patients. Our study showed that CT-severity scores that decreased during re-infection suggest radiological evidence of post-infection immunity against severity despite poor immunity against re-infection especially after 6 months.

## Data Availability

The datasets used and/or analyzed during the current study are available from the corresponding author on reasonable request.

## Abbreviations

COVID-19: Corona virus disease 2019
CT: Computed tomography
CTPA: : Computed tomography pulmonary angiography
GGO: Ground glass opacity
MSCT: Multislice computed tomography
PCR: Polymerase chain reaction
RNA: Ribonucleic acid
RT-RNA: Recombinant ribonucleic acid
SARS-Cov-2: Severe acute respiratory syndrome
SPSS: Statistical package forth social sciences
UK: United Kingdom
